# Eicosanoids, prostacyclin and cyclooxygenase in the cardiovascular system

**DOI:** 10.1111/bph.14167

**Published:** 2018-04-14

**Authors:** Jane A Mitchell, Nicholas S Kirkby

**Affiliations:** ^1^ Cardiothoracic Pharmacology National Heart and Lung Institute London UK

## Abstract

Eicosanoids represent a diverse family of lipid mediators with fundamental roles in physiology and disease. Within the eicosanoid superfamily are prostanoids, which are specifically derived from arachidonic acid by the enzyme cyclooxygenase (COX). COX has two isoforms; COX‐1 and COX‐2. COX‐2 is the therapeutic target for the nonsteroidal anti‐inflammatory drug (NSAID) class of pain medications. Of the prostanoids, prostacyclin, first discovered by Sir John Vane in 1976, remains amongst the best studied and retains an impressive pedigree as one of the fundamental cardiovascular protective pathways. Since this time, we have learnt much about how eicosanoids, COX enzymes and prostacyclin function in the cardiovascular system, knowledge that has allowed us, for example, to harness the power of prostacyclin as therapy to treat pulmonary arterial hypertension and peripheral vascular disease. However, there remain many unanswered questions in our basic understanding of the pathways, and how they can be used to improve human health. Perhaps, the most important and controversial outstanding question in the field remains; ‘how do NSAIDs produce their much publicized cardiovascular side‐effects?’ This review summarizes the history, biology and cardiovascular function of key eicosanoids with particular focus on prostacyclin and other COX products and discusses how our knowledge of these pathways can applied in future drug discovery and be used to explain the cardiovascular side‐effects of NSAIDs.

**Linked Articles:**

This article is part of a themed section on Eicosanoids 35 years from the 1982 Nobel: where are we now? To view the other articles in this section visit http://onlinelibrary.wiley.com/doi/10.1111/bph.v176.8/issuetoc

**Abbreviations**
ADMA: asymmetric dimethyl arginine
BCL‐6: B‐cell lymphoma 6 protein
cPLA_2_: cytosolic phospholipase A_2_
CREB: cAMP response element‐binding protein
EPAC: exchange protein directly activated by cAMP
iPLA_2_: calcium‐independent phospholipase A_2_
NFAT: nuclear factor of activated T cells
NSAID: non‐steroidal anti‐inflammatory drug
PGX: prostaglandin X (prostacyclin)
sPLA_2_: secretory phospholipase A_2_

## Introduction

Eicosanoids and the COX enzyme pathway feature in almost all aspects of health and disease. Indeed, it is difficult to find an organ system, homeostatic process or disease in which COX is not involved. COX has two isoforms; COX‐1 is constitutively expressed in many cell types whereas COX‐2 is constitutively expressed only in certain regions but is readily induced in inflammation and cancer. Arguably the most studied area of COX biology concerns inflammation and pain and this is not surprising as COX‐2 is the therapeutic target for nonsteroidal anti‐inflammatory drugs (NSAIDs), which are amongst the most commonly taken medications worldwide. Indeed, ibuprofen and aspirin, two members of the NSAID family, along with paracetamol, which is not an NSAID but exerts its analgesic effects by inhibiting COX, are members of the WHO Model List of Essential Medicines. However, the importance of COX to the cardiovascular system, particularly within blood vessels, cannot be overstated. This is illustrated by a PubMed search of the term ‘COX’ that delivers 56 082 returns, of these publications 9006 return with the terms ‘COX’ and ‘vascular’. This compares well with the 11 384 publications returning with the terms ‘COX’ and ‘inflammation’. The importance of prostanoids within the cardiovascular system was realized in the remarkable discovery by Sir John Vane in 1976 of a new member of the family made specifically by blood vessels, named at the time, PGX (Moncada *et al.,* 1976). PGX, renamed prostacyclin, is a potent inhibitor of platelet aggregation and a vasodilator; both features of a cardioprotective hormone. The joint discoveries of prostacyclin and the mechanism of action of NSAIDs earned John Vane a share in the Nobel Prize for Physiology and Medicine in 1982. Since its discovery, major advances in our understanding of the importance of prostacyclin have been made. Its structure has been harnessed to produce a class of drugs to treat peripheral vascular disease and pulmonary arterial hypertension and the role its deficiency plays in cardiovascular disease appreciated. Nevertheless, there remains much that we do not know and the full potential of prostacyclin as a biomarker and therapeutic tool has yet to be realized. However, the most pressing issue relating to our understanding of cardiovascular prostacyclin biology is to solve the problem of the much reported cardiovascular side‐effects caused by inhibiting cardio‐protective COX‐2 with NSAIDs. This review focuses on the role of the eicosanoid sub‐group, prostanoids and COX within the cardiovascular system with particular emphasis on prostacyclin and the issue of cardiovascular side‐effects caused by NSAIDs.

## Vascular eicosanoid biology

### Eicosanoids

**Figure 1:**
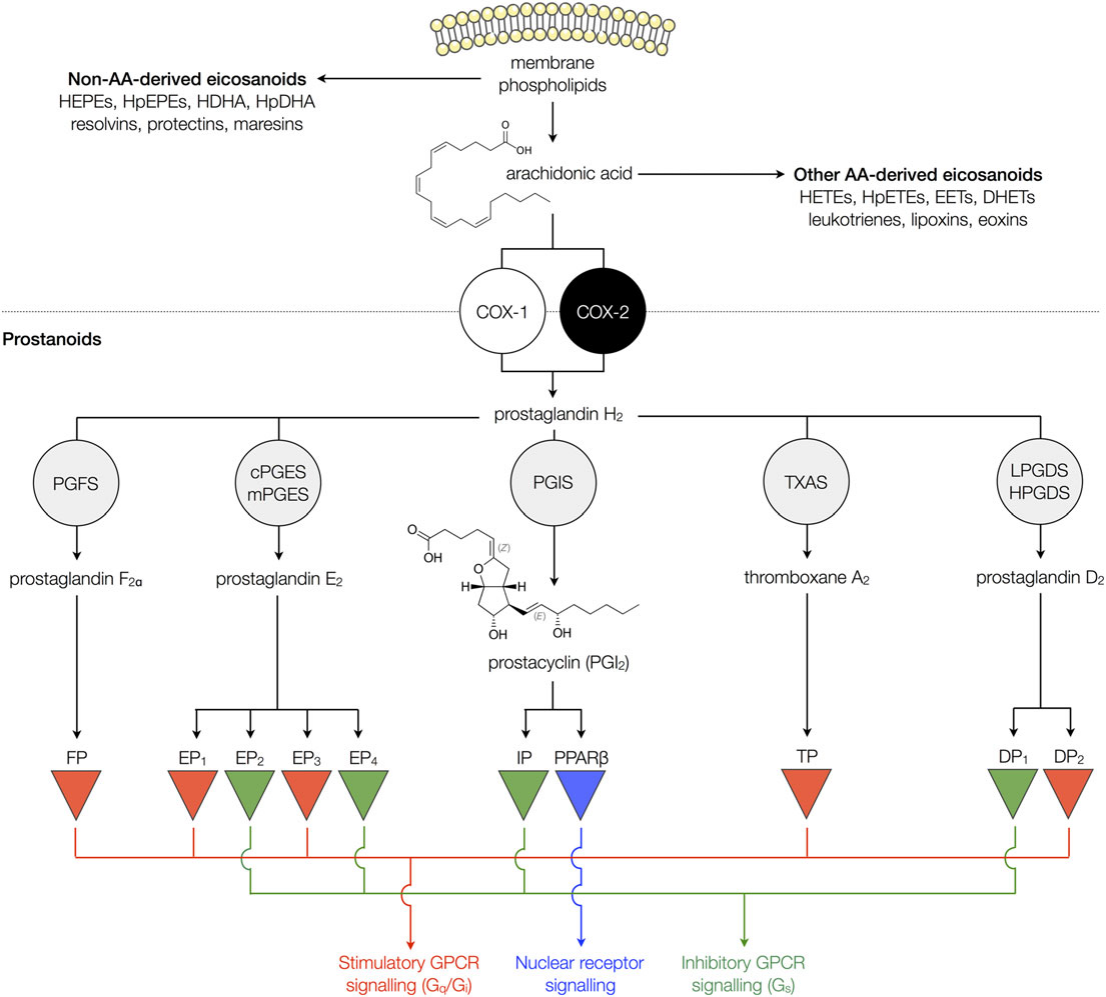
Eicosanoid and prostanoid synthesis. Arachidonic acid (AA) is liberated from membrane phospholipids by the action of PLA_2_. This can be converted to a range of eicosanoid mediators including prostanoids, hydroxyeicosatetraenoic acids (HETEs), hydroperoxyeicosatetraenoic acids (HpETEs), epoxyeicosatrienoic acids (EETs), dihydroxyeicosatrienoic acids (DHETs), leukotrienes, lipoxins and eoxins. Other eicosanoids are formed from other 20 carbon fatty acid substrates, including hydroxyeicosapentaenoic acids (HEPEs), hydroperoxyeicosapentaenoic acids (HpEPEs), hydroxydocosahexaenoic acids (HDHAs), hydroperoxydocosahexaenoic acids (HpDHAs), resolvins, protectins and maresins. For prostanoid synthesis, AA is converted to PGH_2_ by COX‐1 or COX‐2 enzymes. PGH_2_ is then further metabolized to individual prostanoid mediators by specific synthase enzymes including PGF synthase (PGFS), microsomal PGE synthases (mPGES)‐1 and ‐2, cytosolic PGE synthase (cPGES), prostacyclin synthase (PGIS), Tx synthase (TXAS), lipocalin‐type PGD synthase (LPGDS) and haematopoietic‐type PGD synthase (HPGDS). Each prostanoid acts preferentially on its corresponding receptors to activate inhibitory or stimulatory GPCRs or the nuclear receptor, PPARβ.

Eicosanoids are biologically active molecules made by oxidation of either ω‐3 or ω‐6 20‐carbon fatty acids. Eicosanoids are grouped into subcategories including leukotrienes, lipoxins, hydroxy‐eicosatetraenoic acids, hydroxy‐eicosapentaenoic acids, eoxins, isoprostanes, resolvins and prostanoids (Figure 1). Although other members of the eicosanoid family may have vascular effects (Pratico and Dogne, 2009), such as the isoprostane, 8‐isoPGF2α, which contracts vessels by acting on the thromboxane prostanoid TP receptor (Jourdan *et al.,* 1997) or leukotrienes that stimulate vascular leak, contract vessels and propagates inflammation (Colazzo *et al.,* 2017), in blood vessels the best characterized eicosanoids are the prostanoid family with prostacyclin being the predominant prostanoid released by both healthy and diseased blood vessels (Kent *et al.,* 1981; Cannon, 1984; Kirkby *et al.,* 2013c; Kirkby *et al.,* 2014).

### PLA_2_

Prostanoids are synthesized from the 20 carbon ω‐6 fatty acid arachidonic acid by the action of COX. Prostanoid release within most tissues, including blood vessels, is highly regulated and generally requires a strong stimulation before functionally relevant levels are made. COX is expressed in excess in most cells and thereby is generally not the limiting step. Instead, production is controlled by the availability of arachidonic acid. Due to its highly reactive nature and its sensitivity to oxidation, arachidonic acid is not normally free in cells and is instead stored in membrane phospholipids particularly phosphatidylethanolamine, phosphatidylcholine and phosphatidylinositol. The first step in prostanoid synthesis is therefore liberation of arachidonic acid from the membrane by phospholipases (Figure 1). This is mostly carried out by PLA_2_ enzymes, although some arachidonic acid can be formed by the action of PLC acting on arachidonic acid‐containing diacyl‐glycerols. PLA_2_ biology is complex with more than 30 different isoforms loosely classified as (i) cytosolic PLA_2_ (cPLA_2_), activated by μM concentrations of calcium, (ii) calcium independent (iPLA_2_) or (iii) secreted (sPLA_2_), activated by mM concentrations of calcium (Burke and Dennis, 2009; Murakami *et al.,* 2011; Murakami *et al.,* 2014).

Although there is evidence that sPLA_2_ isoforms and iPLA_2_β (Sharma *et al.,* 2010; Majed and Khalil, 2012) can function in endothelial cells under some conditions, most evidence suggests that cPLA_2_ɑ (also referred to as group IVA cPLA_2_; encoded by the PLA2G4A gene) is the major isoform responsible for the release of arachidonic acid to produce prostanoids, in blood vessels.

This essential role of cPLA_2_ɑ in prostanoid production by cells of the cardiovascular system was confirmed in recent work from our group (Kirkby *et al.,* 2015). We obtained cells from a patient with an extremely rare homozygous loss of function mutation of cPLA_2_ɑ and measured a full array of eicosanoids. Endothelial cells, leucocytes and platelets from this patient generated low or undetectable levels of prostanoids including prostacyclin, TxA_2_ and PGE_2_. The results from this patient are in agreement with an earlier study showing reduced production of eicosanoids by platelets from a patient carrying a compound heterozygous mutation of the same isoform of PLA_2_ (Adler *et al.,* 2008).

### COX

The second step in prostanoid synthesis, oxidation of the free arachidonic acid, is catalysed by the COX enzyme (Figure 1). COX is present in two isoforms, COX‐1 and COX‐2. There are structural differences between COX‐1 and COX‐2 within the active site, but for the conversion of arachidonic acid to prostanoids, the enzymic steps, oxidation forming PGG_2_ followed by peroxidation to yield PGH_2_, are identical for both isoforms. PGH_2_ is subsequently converted by downstream synthase/isomerase enzymes to the full range of prostanoids (Figure 1).

The first COX enzyme (what we now know as COX‐1) was purified in 1977 from sheep vesicular glands and found to be a haem protein of 70 kDa (Hemler and Lands, 1977). Just over 10 years later, this form of COX was sequenced (DeWitt and Smith, 1988) and the full‐length cDNA corresponding to a 2.8 kb mRNA was found to encode a protein of 600 amino acids. The authors had the foresight to comment ‘the availability of a full‐length cDNA clone coding for prostaglandin G/H synthase (COX) should facilitate studies of the regulation of expression of this enzyme and the structural features important for catalysis and for interaction with anti‐inflammatory drugs’ (DeWitt and Smith, 1988). They could not have known at the time that within a year, their work would be used to identify a completely new form of COX. Between 1989 and 1991, three separate groups using different experimental approaches identified a new 4.0 kb mRNA species encoding a similar ‘COX’ protein, also of ≈600 amino acids, which we now know as COX‐2 (Rosen *et al.,* 1989; Kujubu *et al.,* 1991; Xie *et al.,* 1991). The first demonstration of human COX‐2, with full enzymic activity, was performed in 1992 using human endothelial cells stimulated with IL‐1 (Hla and Neilson, 1992).

**Figure 2:**
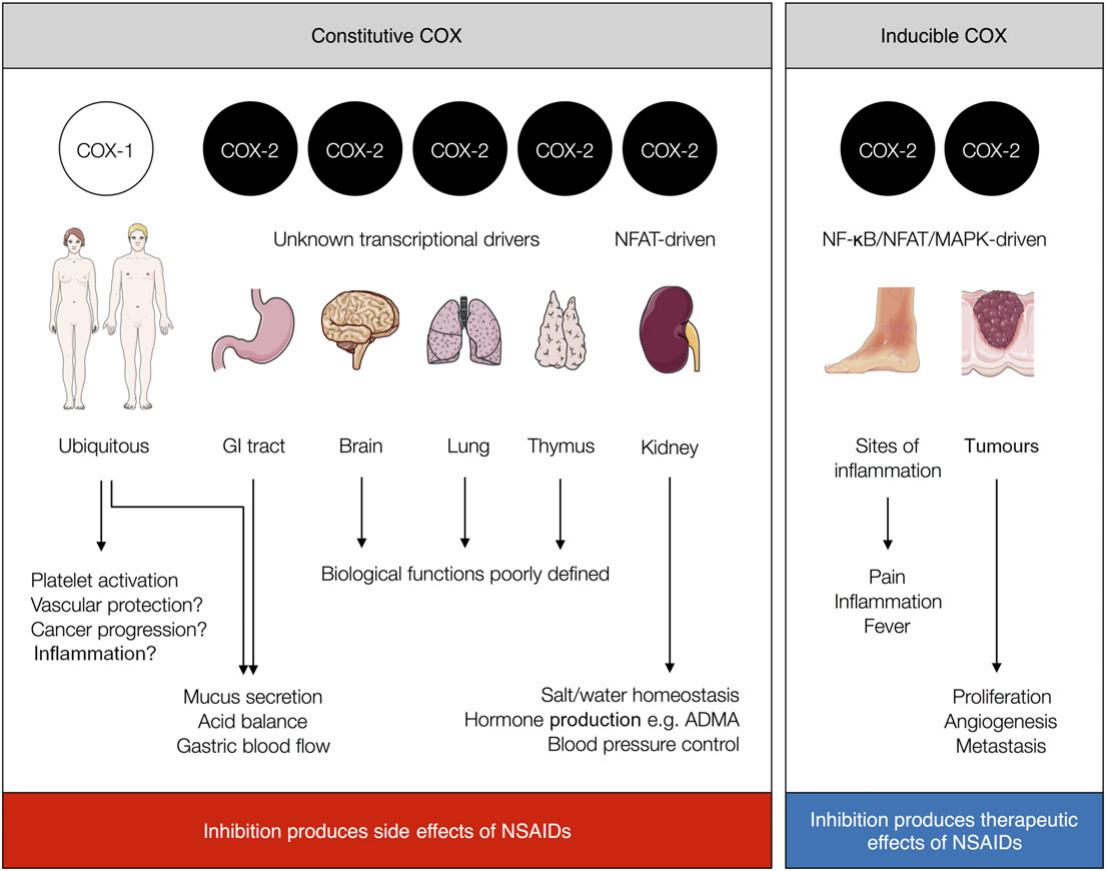
Constitutive and inducible COX enzymes. COX‐1 is a ubiquitous isoform expressed constitutively in all tissues. COX‐2 is constitutively expressed in specific tissue locations within the kidney, gastrointestinal tract, brain, lungs and thymus as well as other regions of the urogenital tract (not shown). Constitutive COX‐2 transcription pathways are known only for the kidney (NFAT). Inducible COX‐2 is expressed at the site of inflammation and in cancer. Inducible COX‐2 transcription pathways include NF‐κB, NFAT and CREB. Inducible COX‐2 is the therapeutic target of the NSAID class of drugs, but their inhibition of constitutive COX‐1 and COX‐2 results in their side‐effects.

A common feature in early studies of COX‐2 was that cells (Rosen *et al.,* 1989; Kujubu *et al.,* 1991; Xie *et al.,* 1991; Hla and Neilson, 1992), blood vessels (Bishop‐Bailey *et al.,* 1997) or laboratory animals (Vane *et al.,* 1994) required an inflammatory insult for the enzyme to be ‘induced’ driven by transiently active transcription pathways including NF‐κB, nuclear factor of activated T cells (NFAT) and cAMP response element‐binding protein (CREB) (Figure 2). Indeed, it was this particular fact that led to the general idea that COX‐1 is constitutively expressed within tissues acting as a protective enzyme whereas COX‐2 is an inducible form present only in inflammation (Figure 2). We now know that this was a simplistic view and that COX‐2 is constitutively expressed in key regions of the body including the brain, lung, thymus, gut and kidney (Kirkby *et al.,* 2013c; Figure 2). In the gut, COX‐2 together with COX‐1 protects the mucosa from injury (Wallace *et al.,* 2000) while, in the kidney, constitutive COX‐2, driven by NFAT, regulates renal function and blood flow. The relevance of renal COX‐2 to cardio‐protection is discussed in detail later. The role of constitutive COX‐2 in the brain, lung and thymus is not completely understood and the transcriptional drivers have not yet been identified.

Early work from our group (Mitchell *et al.,* 1993) and others (Meade *et al.,* 1993) showed that both COX‐1 and COX‐2 were inhibited by NSAIDs but with different potencies, leading us to suggest that COX‐2 is the therapeutic target of NSAIDs and that there was a therapeutic window for the development of COX‐2 selective drugs (Mitchell *et al.,* 1993). Subsequently, COX‐2 selective NSAIDs such as Celebrex® (celecoxib), Vioxx® (rofecoxib) and Arcoxia® (etoricoxib) were introduced for the treatment of inflammation and pain, designed to diminish the gastric side‐effects associated with older style drugs such as diclofenac and ibuprofen. However, because neither the selective COX‐2 inhibitors nor the older NSAIDs discriminate between blocking the COX‐2 induced at the site of inflammation and the constitutively expressed COX‐2 that protects the cardiovascular system, they cause cardiovascular side‐effects. Indeed, understanding and solving the problem of cardiovascular side‐effects caused by NSAIDs remains amongst the most important unresolved questions in the vascular biology of eicosanoids. As such, the remainder of this review will focus on mechanisms of cardiovascular protection by COX‐2 and the involvement of specific eicosanoid and other mechanistic pathways in this aspect of COX‐2 biology.

## Role of COX‐2 in cardiovascular protection and the cardiovascular side‐effects of NSAIDs

### Scale of the problem

Cardiovascular side‐effects, which include hypertension, heart attacks and strokes, were initially thought to be limited to drugs that selectively targeted COX‐2, such as rofecoxib and celecoxib, introduced in the early 2000s. However, as a result of subsequent epidemiology analyses (Warner and Mitchell, 2008; McGettigan and Henry, 2011) and, most recently, the publication of two large clinical cardiovascular outcome studies, SCOT (MacDonald *et al.,* 2017) and PRECISION (Nissen *et al.,* 2016), it is clear that traditional NSAIDs, including ibuprofen and naproxen, carry at least a great cardiovascular risk as the COX‐2 selective drug celecoxib, with ibuprofen emerging as significantly more toxic to the kidney at therapeutic doses (Nissen *et al.,* 2016). Aspirin is a special case in the NSAID family of drugs since it prevents rather than precipitates cardiovascular events. The mechanisms by which aspirin protects the cardiovascular system have been reviewed elsewhere (Crescente *et al*., 2019) but involve the unique pharmacology of aspirin as an irreversible inhibitor of COX which allows this NSAID to selectively target TXA_2_ release from the anucleate platelet. Thus, all NSAIDs, except aspirin, increase personal risk of a cardiovascular event by as much as 30% (McGettigan and Henry, 2011; Trelle *et al.,* 2011; Coxib *et al.,* 2013; Bally *et al.,* 2017). Importantly, increased risk of cardiovascular events while on NSAIDs can be seen after as little as 2 weeks of regular use (Bally *et al.,* 2017). To put this into context, there are ≈460 000 myocardial infarcts and strokes in the UK each year and an estimated 20–30% of the population regularly use NSAIDs. On this basis, the predicted increased risk of a cardiovascular adverse reaction while taking NSAIDs translates to a conservative estimate of around 30 000–50 000 NSAID‐attributable cardiovascular events per year or 10% of all myocardial infarcts and strokes.

The fear of adverse cardiovascular events caused by NSAIDs has led to a series of changes in drug regulation, including (i) the withdrawal of rofecoxib in 2004, (ii) the introduction of ‘black box’ warnings in 2005 and (iii) the reclassification of the over‐the‐counter medication diclofenac as prescription only in 2015 (UK).

Concern regarding cardiovascular events caused by NSAIDs has also resulted in the cautious prescribing of COX‐2 selective drugs (Scarpignato *et al.,* 2015) in favour of the older NSAIDs that are more toxic to the gut or opioids, which carry their own different problems of tolerance and abuse, creating additional health and social burdens. This situation defies logic as we now know that ibuprofen, which is the most consumed NSAID and is still widely available without prescription, causes both cardiovascular side‐effects and is damaging to the gut.

Finally, it is important to remember that NSAIDs are effective chemo‐preventative therapies for cancer, with population‐based studies showing their long‐term use was associated with a 30–60% decrease in the risk of developing major cancers (Matos and Jordan, 2015), with colon cancer being the most extensively studied. Indeed, it should be noted that for colon cancer, evidence is not restricted to epidemiological data but backed up with randomized, placebo‐controlled clinical trials showing that rofecoxib (Baron *et al.,* 2006) and celecoxib (Bertagnolli *et al.,* 2006) were effective in preventing around 50% of tumour occurrence in some patient groups. Taking just this form of cancer as an example, with 43 000 new cases of colon cancer diagnosed in the UK each year, the use of COX‐2 inhibitors has the potential to prevent cancer in 22 000 of these cases annually. Clearly, the potential for NSAIDs to prevent other cancers adds additional importance to assessment of the role of these drugs in population health. However, since the withdrawal of Onsenal (celecoxib) for the prevention of cancer in 2011 because of safety concerns, including cardiovascular side‐effects, NSAIDs are no longer used to prevent cancer.

### Prostacyclin as the major cardioprotective eicosanoid

Of the many eicosanoids produced by the vasculature, prostacyclin is the one most associated with cardiovascular protection and is centrally involved in the mechanisms by which NSAIDs cause cardiovascular side‐effects. As such, its production and signalling are discussed below.

**Figure 3:**
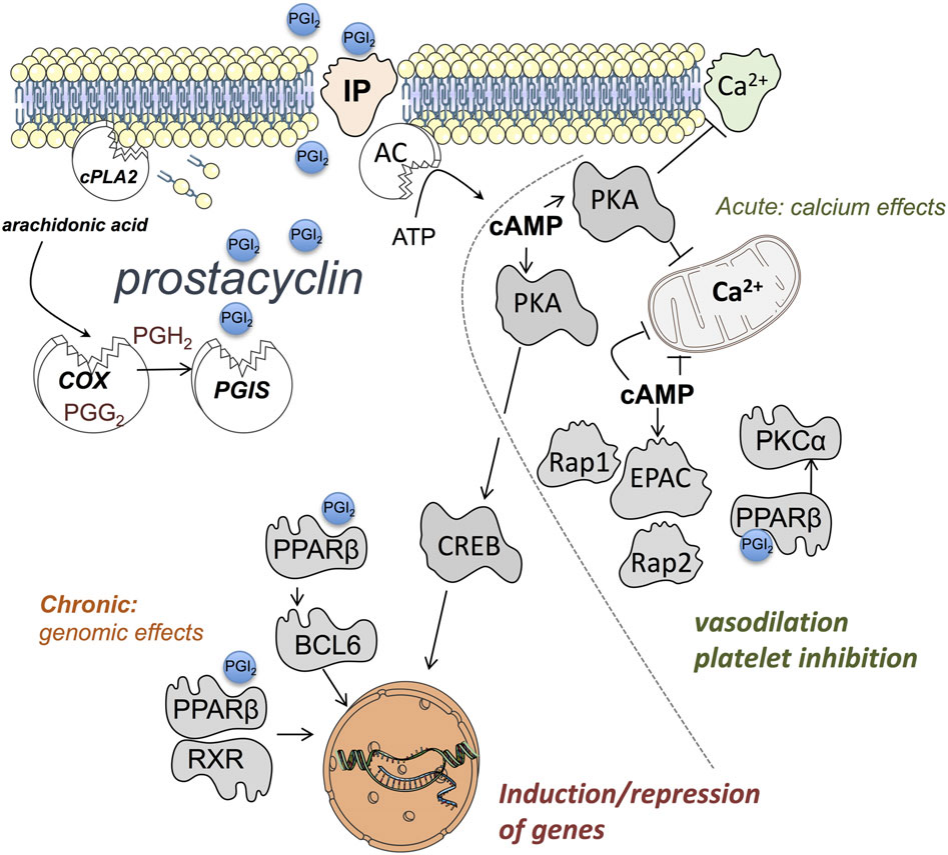
Synthesis and signalling pathways for prostacyclin (PGI_2_). Arachidonic acid is released by the actions of cytosolic PLA_2_ (cPLA_2_) then metabolized to prostacyclin by the concerted actions of COX and prostacyclin synthase (PGIS). Prostacyclin acts on cell surface IP receptors linked to action of adenylate cyclase (AC) resulting in the conversion of ATP to cAMP. cAMP then activates (i) PKA which phosphorylates substrate proteins leading to blockade of calcium channels and of release from intracellular stores. cAMP activated PKA also activates the transcription factor cAMP response element binding protein (CREB) leading to gene induction. cAMP also activates exchange protein directly activated by cAMP (EPAC), a Ras activate protein (Rap)1 and Rap2 guanine‐nucleotide‐exchange factor. Activated EPAC also prevents calcium elevation within cells (by unknown mechanisms). Prostacyclin also activates cytosolic PPARβ receptors that function in two ways; (i) via classical PPAR‐retinoid X receptor complex signalling to modulate gene expression and (ii) by binding and repressing the transcription factor BCL6 (inactive PPARβ) and PKCα, (active PPARβ). These pathways orchestrate acute signalling events characterized by calcium reduction leading to (for example) vasodilation and inhibition of platelet activation and chronic events mediated by gene induction.

The reason why blood vessels release predominantly prostacyclin is because they are enriched with both COX and prostacyclin synthase (CYP8A1; Figure 3). Prostacyclin synthase was first purified from bovine aorta (DeWitt and Smith, 1983). Although prostacyclin synthase knockout mice have been developed, surprisingly, they have not been extensively used in cardiovascular models. A single study describes prostacyclin synthase knockout mice as having altered renal developmental and renal dysfunction, increased BP and, consequently, vascular hypertrophy (Yokoyama *et al.,* 2002). Perhaps, more informative, human mutations and polymorphisms in prostacyclin synthase are linked with essential hypertension, myocardial and cerebral infarction (Nakayama, 2010).

Although the cardioprotective pedigree of prostacyclin is well established, apart from its effects on platelets, which are (i) inhibitory, (ii) highly reproducible across models and (iii) independent of platelet activation pathways, the effects of prostacyclin can be subtle and influenced by the resting state of the target cell or organ. This is because prostacyclin signalling utilizes different types of receptors and transduction pathways (Figures 1 and 3). For example, prostacyclin can exert acute effects, such as inhibition of platelet activation and relaxation of blood vessels, which are evident within seconds or minutes of adding prostacyclin to the system. Prostacyclin can also exert longer term genomic effects by directing gene transcription. Prostacyclin acts primarily on two receptors; the cell surface GPCR IP receptor and the cytosolic nuclear receptor PPARβ (Mitchell *et al.,* 2014). Additionally, in some situations, prostacyclin can act on other prostanoid receptors to produce common and/or contradictory effects. This aspect of prostacyclin signalling has been reviewed in detail elsewhere (Luo *et al.,* 2016).

IP receptors are linked to activation of membrane‐bound adenylate cyclase, which converts ATP to the intracellular signalling molecule cAMP. cAMP then activates PKA, engaging the ‘PKA pathway’ and/or exchange protein directly activated by cAMP (EPAC), engaging the ‘EPAC pathway’. These pathways, reviewed in detail elsewhere (Torres‐Quesada *et al.,* 2017; Wang *et al.,* 2017), are complex and incompletely understood. For vessels to contract or platelets to aggregate, intracellular calcium must be increased. Activation of PKA and/or engagement of EPAC in platelets or vascular smooth muscle cells results in immediate and profound reduction in levels of intracellular calcium through incompletely understood pathways (Yan *et al.,* 2016).

However, for PKA, at a very basic level, activation results in phosphorylation of its substrate proteins (of which there are many) (Shabb, 2001); including those that regulate intracellular calcium handling and mediate contraction or aggregation. This leads to (i) depletion and/or blockade of release from intracellular calcium stores, (ii) inhibition of subsequent store‐operated calcium entry (Cuinas *et al.,* 2016), (iii) blockade of calcium entry from voltage‐gated (and other) calcium channels (Shabb, 2001) and activation of MaxiK and ATP sensitive potassium channels and (iv) reduced sensitivity of effector proteins such as myosin light chain kinase that mediate contraction/aggregation.

Less is known about the EPAC pathway. There are two EPAC isoforms, EPAC1 and EPAC2 (Lezoualc'h *et al.,* 2016), both of which are guanine‐nucleotide exchange factors for the Ras‐like GTPases, Rap1 and Rap2. EPAC mediates vasodilation by indirectly regulating calcium‐sensitive and ATP‐sensitive potassium channels and by lowering contractility by inhibition of small GTPase RhoA activity (Lezoualc'h *et al.,* 2016). Although Rap1 and Rap2 signalling is known to be involved in platelet adhesion (Stefanini and Bergmeier, 2016), the role of EPAC in prostacyclin signalling in platelets has not been addressed. The chronic effects of IP receptor activation can be explained in part by PKA‐dependent activation of the transcription factor CREB. CREB binds to the CRE sequences in DNA to orchestrate transcriptional changes of ≈4000 target genes in the human genome (Zhang *et al.,* 2005), although how this relates to specific responses associated with IP receptor activation within cardiovascular structures remains to be fully explored.

Work from our group and of others has shown that activation of PPARβ can also explain some of the acute effects of prostacyclin in platelets and blood vessels (Ali *et al.,* 2009a; Baron *et al.,* 2006; Harrington *et al.,* 2010; Li *et al.,* 2012; Kirkby *et al.,* 2018). Initially, this was a surprising discovery as PPARβ was thought to be a pathway that regulates gene induction rather than acute signalling events; however, now we know that PPARβ activation leads to the engagement of both genomic and non‐genomic pathways (Mitchell *et al.,* 2014). First, activation of PPARβ can act via classical PPAR‐retinoid X receptor signalling to regulate target gene induction. Target genes of PPARβ include ANGPTL4 and a range of lipid metabolism and angiogenic genes (Kojonazarov *et al.,* 2013). The angiogenic profile of PPARβ explains in part why selective agonists of this receptor can induce cancer and provides a mechanism by which prostacyclin drives proliferation in some cells including endothelial progenitor cells (He *et al.,* 2008). Second, in its resting state, PPARβ binds and represses the transcription factor BCL‐6 (Lee *et al.,* 2003) and, in its active state, binds the protein kinase PKCα (Ali *et al.,* 2009b; Mitchell *et al.,* 2014). Consequently, when prostacyclin binds PPARβ, BCL‐6 is released, and PKCα is repressed to regulate inflammatory gene transcription and the acute effects seen in platelet activation respectively. It remains unclear precisely how PPARβ agonists induced acute vasodilation (Toral *et al.,* 2017), although this may involve the RhoA pathway (Harrington *et al.,* 2010).

While there is much we do not yet know about prostacyclin and its signalling pathways, drugs acting on prostacyclin receptors have been developed and used, for example, in the treatment of pulmonary arterial hypertension (see Mitchell *et al.,* 2014). Considering the powerful effects of prostacyclin in the cardiovascular system, it is likely that prostacyclin has further potential as a therapy for more common forms of cardiovascular diseases, and this may come from a full understanding of its receptor and signalling pathways. However, to solve the problem of cardiovascular side‐effects caused by NSAIDs, we also need to understand how *endogenous* prostacyclin works to protect the cardiovascular system.

### Mechanisms of NSAID cardiovascular side‐effects

When COX‐2 was discovered as an isoform of COX induced at the site of inflammation, it was known that COX‐1 and prostacyclin synthase were both constitutively expressed in endothelium. Thus, based on what was known at the time, studies showing that COX‐2 induced in vessels produced prostacyclin (Mitchell and Evans, 1998) and that rofecoxib or celecoxib selectively reduced urinary prostacyclin metabolites (Catella‐Lawson *et al.,* 1999), raised concerns about the cardiovascular safety of COX‐2 inhibitors before clinical data were available. In this regard, the field was ‘primed’ and alerted to the possibility that COX‐2 selective NSAIDs might cause cardiovascular side‐effects. In 2000, when results of the VIGOR study (Bombardier *et al.,* 2000) showed higher numbers of heart attacks in people taking rofecoxib than the comparator drug, naproxen, serious debate about cardiovascular side‐effects began. However, it was only after the publication of the APPROVe trial (Bresalier *et al.,* 2005) where rofecoxib was tested against placebo and showed increased cardiovascular events that this became a widely accepted side‐effect and resulted in the withdrawal of the drug worldwide.

Despite almost 20 years of research in the area, we still do not fully understand how COX‐2 protects the cardiovascular system. It is generally agreed that prostacyclin is the major cardio‐protective prostanoid, but an important limitation in the field has been separating the respective roles of COX‐1 and COX‐2, in prostacyclin production and, furthermore, to determine the location of cardio‐protective COX‐2. It is now believed that cardio‐protective COX‐2 is located in the kidney and/or the systemic vascular endothelium. Although there is debate regarding the importance of homeostatic COX‐2 between these locations, it is known that *constitutively* expressed COX‐2 (Cheng *et al.,* 2006; Ahmetaj‐Shala *et al.,* 2015; Kirkby *et al.,* 2016), rather than an inflammation‐driven isoform protects the cardiovascular system. This understanding is based on the findings that (i) NSAID‐associated cardiovascular events occur in patients, without overt cardiovascular disease or systemic inflammatory disease and (ii) blocking COX‐2 in healthy people or mice impairs renal function (Whelton and Hamilton, 1991; Ahmetaj‐Shala *et al.,* 2015) and increases BP (Ahmetaj‐Shala *et al.,* 2015) and thrombosis (Yu *et al.,* 2012) in otherwise healthy laboratory animals.

### Role of COX‐1 and COX‐2 in vascular prostacyclin production

**Figure 4:**
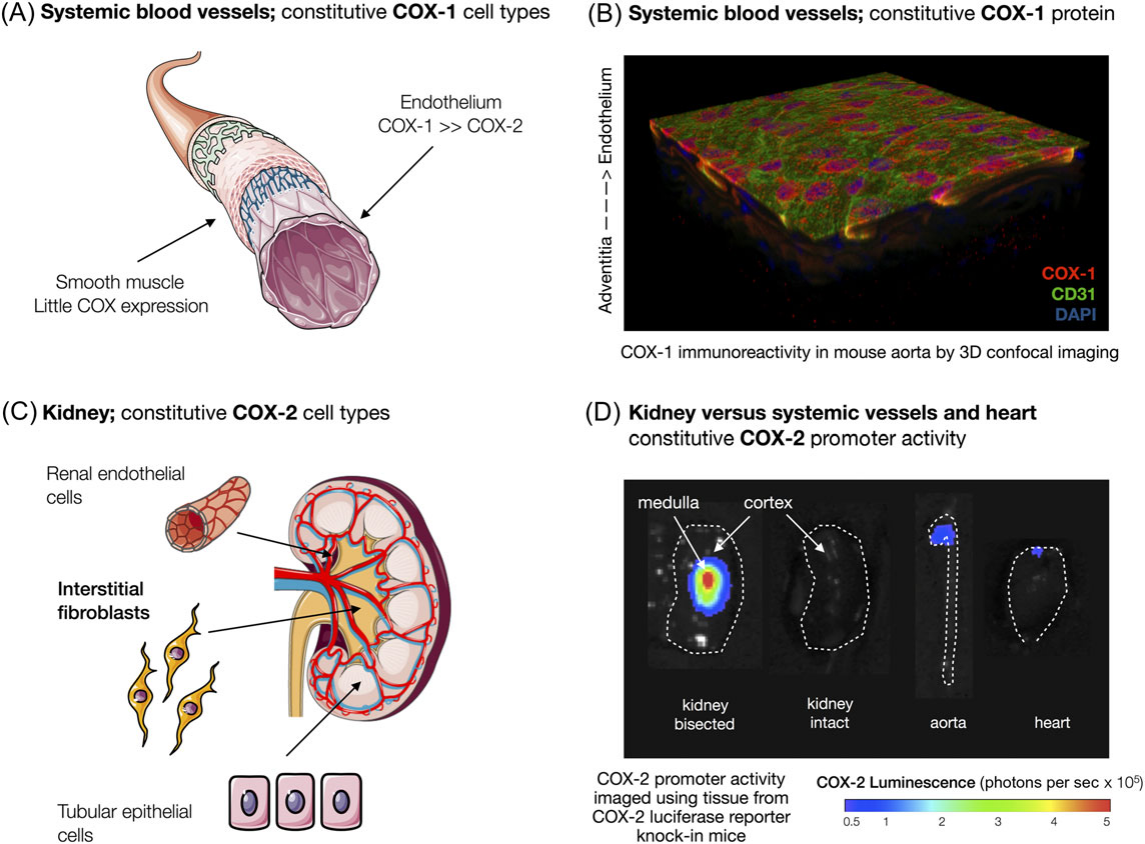
Expression of COX‐1 and COX‐2 at systemic vascular and renal sites. COX‐1 expression predominates over COX‐2 in the endothelium of systemic vessels (A) shown as red staining, with nuclei stained blue and CD31 stained green (B). COX‐2 is expressed in high levels within particular cells within the kidney including the specialized fibroblasts within the interstia of the renal medulla, renal tubule cells and the endothelium of renal blood vessels (C). COX‐2 gene expression is visualized as an intense area of activity within the bisected kidney that maps to the inner renal medulla where levels are very much higher than in systemic blood vessels or the heart (D).

The case for COX‐2 expressed in the systemic vascular endothelium producing prostacyclin is based on two lines of indirect research. The first is that a metabolite of prostacyclin, PGI‐M, which serves as a urinary marker of this prostanoid, is decreased in subjects taking COX‐2 selective NSAIDs (McAdam *et al.,* 1999). However, the idea that *urinary* markers of prostacyclin reflect levels in the *systemic* circulation is not universally accepted (Mitchell and Warner, 2006; Flavahan, 2007) and current evidence suggests that urinary PGI‐M is, in fact, a renal metabolite, i.e. it reflects more *renal* levels, rather than *systemic* levels of prostacyclin (Mitchell, 2016; Kirkby *et al.,* 2017a; Mitchell *et al.,* 2017). The second line comes from studies showing that endothelial COX‐2 gene deletion in mice causes thrombosis (Yu *et al.,* 2012). However, these data and reported phenotypes may also be explained by the loss of COX‐2 locally expressed in the renal rather than in systemic vascular endothelium. Further, the idea that COX‐2 is the major determinant of prostacyclin levels in the systemic circulation does not take account of research showing the importance of COX‐1 in prostacyclin production. COX‐1 and prostacyclin synthase are both constitutively expressed in endothelial cells (Figure 4). Consequently, blood vessels from COX‐1 knockout mice, stimulated with calcium ionophore to activate PLA_2_, release negligible levels of prostacyclin (Kirkby *et al.,* 2012; Liu *et al.,* 2012; Kirkby *et al.,* 2013c; Luo *et al.,* 2016) whereas prostacyclin release is essentially unchanged in freshly prepared arteries from COX‐2 knockout mice (Kirkby *et al.,* 2012; Kirkby *et al.,* 2013c). Similarly, plasma levels of prostacyclin are reduced in COX‐1 knockout mice but unchanged in mice where COX‐2 is deleted (Kirkby *et al.,* 2012; Kirkby *et al.,* 2013c). Most recently, our data using novel, tissue‐specific, COX knockout mice have confirmed that in systemic blood vessels, COX‐1 in endothelial cells accounts entirely for prostacyclin release from endothelium (Mitchell *et al.,* 2017). This is also the case for vessels from atherosclerotic mice where although COX‐2 is expressed locally in lesions, its role in prostacyclin release in comparison with COX‐1 is negligible (Kirkby *et al.,* 2014). In fact, to model a situation experimentally in mice where COX‐2 takes over from COX‐1 as the major source of prostacyclin production, severe systemic inflammation needs to be induced (Kirkby *et al.,* 2013a). Clearly then, *in vivo*, and in defined blood vessels *ex vivo*, COX‐1 and not COX‐2 determines prostacyclin production. While this conclusion is not universally accepted, wherever investigated at the protein, gene or bioactivity level, COX‐2 is sparse, compared with COX‐1, in the majority of endothelial sites. To explain this, it has been suggested that COX‐2 in endothelial cells is induced by shear stress and is very unstable outside the body (Funk and FitzGerald, 2007). This suggestion has been addressed in experiments using fresh vessels and by showing that in plasma, basal as well as bradykinin‐induced prostacyclin release *in vivo* is COX‐1, and not COX‐2 dependent (Kirkby *et al.,* 2012). Most recently, it has been suggested that low levels of COX‐2 activity can still contribute importantly to prostacyclin production, because this isoform requires lower levels of arachidonic acid for activation compared with those required by COX‐1 (Grosser *et al.,* 2017). However, any small differences in the enzyme kinetics do not adequately explain the overwhelmingly dominant role of COX‐1 over COX‐2 in the control of basal plasma prostacyclin levels (Kirkby *et al.,* 2012; Kirkby *et al.,* 2013c) and *ex vivo* vascular prostacyclin production over a range of stimuli or substrate levels (Kirkby *et al.,* 2013c).

### Role of the kidney in cardiovascular protection by COX‐2

By contrast to the lack of clear, direct evidence for a role of COX‐2 in vascular prostacyclin production, the alternative case for a role for renal COX‐2 in cardiovascular protection is supported by a wealth of data. This includes studies which have shown COX‐2 as being constitutively expressed in interstitial fibroblasts in the renal medulla, tubular epithelial cells and renal endothelial cells (Harris, 2006) (Figure 4). Our recent work (Kirkby *et al.,* 2016) confirms the medullary fibroblast region as a major site for COX‐2 and shows that expression is driven by the NFAT transcription factor, in the absence of inflammation. At a functional level, constitutive COX‐2 regulates fundamental aspects of renal homeostasis, including renin release, sodium excretion, renal blood flow and BP (see Harris, 2006). For example, whereas COX‐2 knockout mice exhibit negligible changes in the transcriptome of the aorta, heart or blood, such deletion of COX‐2 produces a profound effect on the renal transcriptome (>1000 genes) directly demonstrating the importance of COX‐2 in the kidney, relative to other cardiovascular structures (Ahmetaj‐Shala *et al.,* 2015). Consequently, the effects of NSAIDs in the kidney may explain the cardiovascular side‐effects caused by inhibitors of COX‐2.

### Role of eNOS in COX‐2 cardiovascular protection

**Figure 5:**
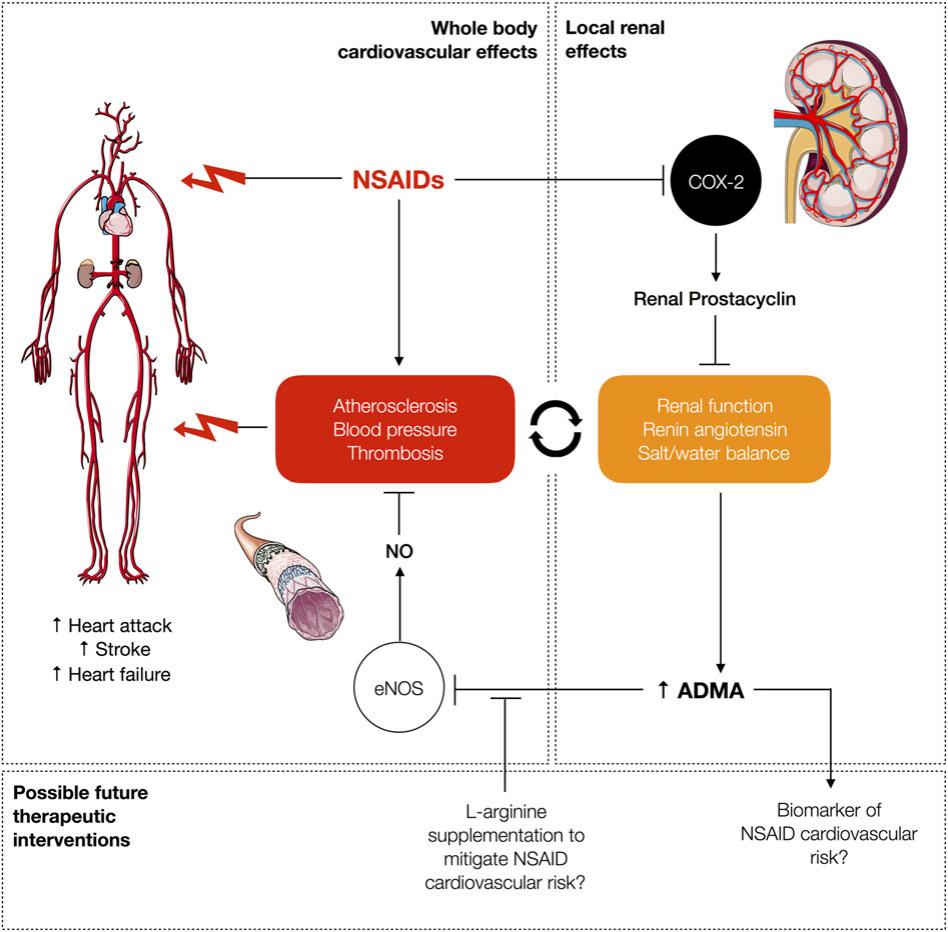
Hypothesis: pathways that link inhibition of renal COX‐2 prostacyclin with endothelial NOS (eNOS) inhibition and increased risk of cardiovascular events. Blocking COX‐2 with NSAIDs in the kidney results in increased levels of ADMA in the systemic circulation, where it inhibits eNOS. Inhibition of eNOS increases atherosclerosis, hypertension and thrombosis. Blocking COX‐2 in the kidney also decreases renal function and increases the renin‐angiotensin and sodium retention pathways. The eNOS‐dependent systemic effects and local effects within the kidney, work together to amplify the cardiovascular dysfunction and work in concert to predispose people taking NSAIDs to cardiovascular side‐effects.

Until recently, precisely how inhibition of COX‐2 derived prostacyclin resulted in hypertension, thrombosis and atherosclerosis was unclear and not adequately explained by the simple removal of prostacyclin as the sole event. A significant advancement in our understanding of how COX‐2 mediates cardiovascular protection was made in recent work from our group (Ahmetaj‐Shala *et al.,* 2015) and others (Yu *et al.,* 2012) describing a link between inhibition of COX‐2 and the endothelial NOS (eNOS) pathway (Figure 5). Our work (Ahmetaj‐Shala *et al.,* 2015) showed that when COX‐2 is deleted in mice or blocked with NSAIDs in mice or healthy human volunteers, an inhibition of the methylarginine pathway is removed that results in increased levels of the endogenous eNOS inhibitor, asymmetric dimethylarginine (ADMA). We have now repeated, validated and extended these observations to show that the effect of COX‐2 inhibition on ADMA levels is reproduced in mice lacking prostacyclin synthase but not in PGE_2_ synthase knockout mice (Raouf *et al.,* 2016), firmly establishing prostacyclin as the protective COX‐2 end product that limits ADMA levels (Figure 5).

The constitutive isoform, eNOS, is expressed throughout the vasculature where it protects the cardiovascular system against atherosclerosis, thrombosis and hypertension. The effects of eNOS and COX‐2 together in protecting the cardiovascular system is biologically enhanced as NO and prostacyclin work additively to maintain vascular function (Lidbury *et al.,* 1989) and in a synergistic manner against thrombosis (Levin *et al.,* 1982; Macdonald *et al.,* 1988; Lidbury *et al.,* 1989; Kirkby *et al.,* 2013b). This pathway, which links COX‐2 and eNOS, via prostacyclin, provides a plausible mechanism for how NSAIDs could precipitate cardiovascular side‐effects and highlights the circulating levels of ADMA as a potential biomarker of such side‐effects. Finally, because the inhibitory effects of ADMA on eNOS can be prevented by L‐arginine, a simple, safe, nutritional supplement available without prescription, this mechanism also provides a possible rescue therapy for those people at risk who need to take NSAIDs to go about their daily lives (Figure 5).

### Role of genetics in cardiovascular protection by COX‐2 and potential of genetic biomarkers

In addition to classical hypothesis testing studies such as those described above, this field needs hypothesis‐generating approaches. With this in mind, we have performed a first of its kind genome‐wide association study using data from a set of ~1000 samples from participants in the Adenoma Prevention with Celecoxib trial (Solomon *et al.,* 2005) and identified four loci exceeding a nominal discovery *P*‐value threshold of 10^−6^ but not predicting general cardiovascular risk (CARDIOGRAMplusC4D Consortium), thus suggesting they may have a specific, causative role in NSAID cardiovascular toxicity (Kirkby *et al*., 2017b). Two of these loci map to viable candidate genes, AHNAK and NCKX2, which we showed to be functionally relevant in renal vessels from knockout mice (Kirkby *et al*., 2017b). These data, while preliminary, are the first to deliver potential genetic biomarkers of NSAID‐induced cardiovascular adverse events, thereby paving the way for precision medicine in this area.

## Summary and future directions

Prostacyclin is a fundamental cardio‐protective hormone whose therapeutic potential will, as we decipher its signalling pathways, inevitably be realized. NSAIDs are here to stay despite the cardiovascular side‐effects that have blighted their recent history. We need to understand the mechanisms by which these occur in order to protect those at risk and so that COX‐2 inhibitors can be used to prevent cancer. This should include testing the translational potential of targeting the COX‐2/prostacyclin/ADMA/eNOS axis to identify those at risk (by measuring ADMA levels) and to protect those taking NSAIDs (with L‐arginine supplementation). Hypothesis‐testing approaches should be complemented by the integration of genetic data and other ‘‐omic’ approaches to find biomarkers and generate new hypotheses to extend our understanding of COX, prostacyclin and eicosanoids in the cardiovascular system.

### Nomenclature of targets and ligands

Key protein targets and ligands in this article are hyperlinked to corresponding entries in http://www.guidetopharmacology.org, the common portal for data from the IUPHAR/BPS Guide to PHARMACOLOGY (Harding *et al.,* 2018), and are permanently archived in the Concise Guide to PHARMACOLOGY 2017/2018 (Alexander *et al.,* 2017a,b,c).

## Conflict of interest

Within the last 5 years, J.A.M. has acted a consultant and expert witness in cases relating to prostacyclin drugs and non‐steroidal anti‐inflammatory drugs. J.A.M. has received unconditional educational awards from pharmaceutical companies including United Therapeutics and Actelion and is on the scientific advisory board of Antibe Therapeutics. The authors report no other conflicts of interest.
